# Supplementary material for: A Multi-Parameter Analysis of Cellular Coordination of Major Transcriptome Regulation Mechanisms
Source: Sci Rep. 2018 Apr 10;8:5742. doi: 10.1038/s41598-018-24039-1 (PMC5893539; doi:10.1038/s41598-018-24039-1)
Supplement: Supplementary file 1 — Supplementary figures and tables [file 41598_2018_24039_MOESM1_ESM.docx]

**A Multi-Parameter Analysis of Cellular Coordination of Major Transcriptome Regulation Mechanisms**

Wen Jiang (w_jiang@yahoo.com)^a,b^, Zhanyong Guo (gzy@henau.edu.cn)^d^, Nuno Lages (nflages@gmail.com)^e^, W. Jim Zheng (wenjin.j.zheng@uth.tmc.edu)^f^, Denis Feliers (feliers@uthscsa.edu)^g^, Fangyuan Zhang (fangyuan.zhang@ttu.edu)^c^, Degeng Wang (degeng.wang@ttu.edu)^a,b,h^

^a^Department of Environmental Toxicology, ^b^The Institute of Environmental and Human Health (TIEHH), ^c^Department of Mathematics & Statistics, Texas Tech University, Lubbock, TX 79409.

^d^National Key Laboratory of Wheat and Maize Crop Science, College of Agronomy, Henan Agricultural University, Zhengzhou, Henan 450002, P. R. China

^e^Instituto Superior Técnico, Universidade de Lisboa, IST-Taguspark, 2744-016 Porto Salvo, Portugal

^f^School of Biomedical Informatics, The University of Texas Health Science Center at Houston, 7000 Fannin Street, Suite 600, Houston, TX 77030.

^g^Department of Medicine/Renal Diseases, The University of Texas Health Science Center at San Antonio, San Antonio, TX

^h^: Corresponding author. Email: [degeng.wang@ttu.edu](mailto:degeng.wang@ttu.edu).

Keywords: multi-parameter analysis, polysome, post-transcription regulation, transcription rate (TR), untranslated region (UTR)

**Figure Legends**

Figure S1: Illustration of polysome profile. An example polysome profile is shown. The polysomal and non-polysomal fractions are illustrated along the x-axis.

Figure S2: Scatter plot of the stability and the translation indices for mRNAs of the PMS and the LSM functional groups of genes. The PMS mRNAs are shown in red color, and the LSM mRNAs in black color. LSM8 mRNA was denoted.

Figure S1


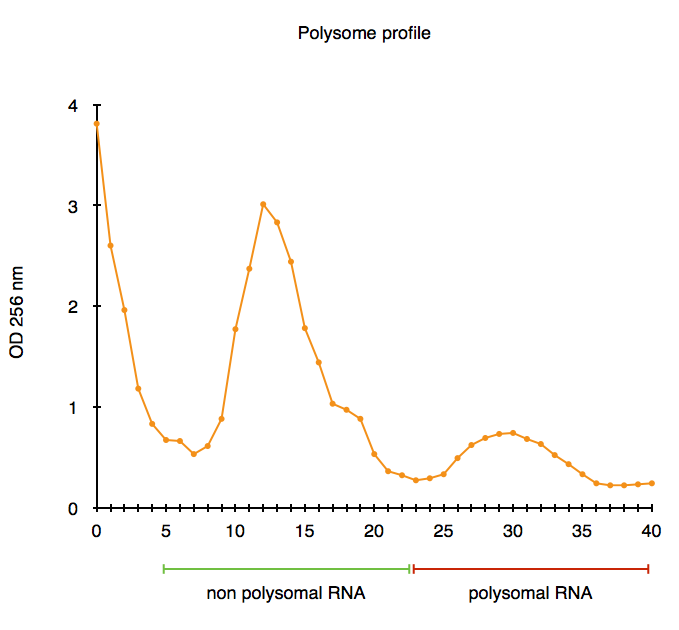


Figure S2

Table S1

| Table S1: Comparison of the statistical features of the profiles of the three parameters. | | | | |
| --- | --- | --- | --- | --- |
|  | Standard Deviation | Mean | Value Range | |
|  |  |  | From | To |
| log_2_(TR) | 1.53 | 3.85 | -2.68 | 10.41 |
| log_2_(RA) | 2.38 | 3.18 | -6.02 | 12.33 |
| log_2_(TA) | 2.77 | 2.74 | -7.18 | 13.04 |

Table S2

| Table S2: Comparison of the UTR proportions of the PSM and the LSM mRNAs | | | |
| --- | --- | --- | --- |
|  | No. of Genes | Median | Mean |
| LSM | 8 | 68.6% | 69.6% |
| PSM | 14 | 26.4% | 35.3% |
| p-value = 0.0004 (two-sample t-test with a “greater than” alternative hypothesis) | | | |
